# Turning over New Leaves

**Published:** 1888-02-25

**Authors:** 


### TURNING OVER NEW LEAVES.

[Books for Review should be sent to The Editor, The Lodge, Porchester Square, W.]

### Thy Heart with My Heart.*

IT has seldom been our fortune to meet with a book which contained so much valuable matter in so little space as this tiny pamphlet. The four letters are written in a spirit of noble and intelligent religion that must commend them to every thoughtful mind. The author is evidently a man of broad and catholic spirit, who has gone to the root of the matter, and places the whole meaning and intention of the Communion as our Lord established it on a basis at once so simple and so high that every reverent and honest mind must at once understand and appreciate it. We refrain from citations from this beautiful little work because we hope to give selections from it in our "Words of Consolation."

### A Prayer-book for Hospitals.†

THIS excellent little volume contains what the author aptly calls "family prayers" for use in hospital wards, besides a service for public worship in the hospital. In both the prayers are well adapted to the special circumstances of those who take part in them, being marked by a very reverent spirit, and carefully avoiding anything in which members of any branch of the Christian Church could not freely join; and while petitions for restoration to health necessarily take a prominent place in them, the whole tone of the prayers is that of cheerful reliance on God's mercy. Morning and evening prayers for a week are given, besides the form for public service, which is also published separately at the price of 1*s.* 6*d.* per dozen copies, for the purpose of being distributed among the worshippers. On one point only do we take exception to Dr. Waring. He suggests in his preface that the time occupied by the daily morning and evening prayers should be half an hour, and that the public worship "should not exceed an hour" in length. We think that, considering that the patients who listen to the first are often very feeble and unable to bear any fatigue of mind and body, while many of those who are present at the public service are only convalescent, half the time specified would be enough in each case. This is, however, a matter which may safely be left to the discretion of those who lead the services, and, in any case, it is a trifling fault in an otherwise excellent work.

### The Care of Common Diseases.*

DR. BASIL's little volume consists of the expansion of a lecture delivered to a popular audience, and so deals with facts familiar to all members of his profession. But these very elementary facts—how to deal at a moment's notice with such alarming accidents as obstructed respiration, sudden hemorrhages of all sorts, burns, sprains, and cuts; as well as the care of the *sequelæ* of typhoid fever, diphtheria, &c.—are for the most part unknown to the public at large; and Dr. Basil puts them before his readers in very straightforward

* "Thy Heart with My Heart." Four simple letters on the Holy Communion. By the Rev. P H. Newnham, M.A., chaplain to the Devon and Exeter Hospital. London: Longmans, Green, and Co.

† "The Hospital Prayer-book, Containing Prayers for Daily and Occasional Use; also a Short Form of Public Service for Lay Readers in Hospitals." By Edward John Waring, C.I.E., M.D., Fellow of the Royal College of Physicians of London, author of "Cottage Hospitals: Their Objects, Advantages, and Management," etc. Second edition, revised and corrected. London: J. and A. Churchill.

* "The Commoner Diseases and Accidents to Life and Limb." By M. M. Basil, M.A., M.B., C M. (Edin.). London: J. and A. Churchill.

style, which is varied occasionally by an amusing anecdote or an apt quotation. The little treatise is thoroughly readable, and the avoidance of technical terms makes it comprehensible to all. Though, as the author says at the outset, it is not intended to enable anyone to do without the doctor, it will give all who read it valuable hints as to the wisest conduct in emergencies till the doctor comes, and may impress upon them the necessity of those precautions with regard to suitable food, pure water, and fresh air, which, if conscientiously followed, would prevent many of the diseases that now swell our bills of mortality.

### A New Cure for Consumption.*

Dr. Weaver claims in this little book to have discovered an absolute cure for consumption—namely, treatment by iodide of potassium, with morphia, and subsequently iron or one of the various preparations of cinchona. He says that he has cured by this means patients who were in an advanced phthisical condition, besides preventing the development of the disease where it was incipient. The author complains that among his fellow-practitioners his method of treatment has not been received with acceptance or even with courtesy, in spite of the proofs he adduces of its success. This complaint we think rather unjust. Medical men are not, as a rule, so sceptical as to refuse consideration to any remedy or palliative which can give a satisfactory account of itself; when the action of the drug is rationally explained they are always ready to give it a fair trial. But on this point Dr. Weaver says nothing. He does not tell his readers how the iodide of potassium restores the lung tissue which has been destroyed by the disease, or prevents the further development of tubercles. A medical man, writing ostensibly for medical men, should not omit this; we cannot accept a treatment and apply it to patients on the Dr. Fell principle. We have a right to know "the reason why," and Dr. Weaver's reticence on this point may very reasonably explain the attitude he says other doctors adopt towards him. The volume concludes with a eulogistic chapter on Southport, where the author practises, as a health resort.

### Surgery for Nurses.†

A work from the pen of a clinical teacher so popular and so distinguished for lucidity and vividness as Dr. Joseph Bell is sure to be valuable, and this little volume, with its clear and concise directions for the conduct of surgical cases, might advisably be included in every nurse's library. It is not meant for the general public, though its admirable clearness in explaining the meaning of technical expressions, and in stating every fact and explaining every symptom in the plainest possible language, make it perfectly comprehensible to anyone. But being the substance of lectures given to the nurses at Edinburgh Infirmary during twelve successive years, it goes on the assumption that those who read, as those who heard its contents are women sufficiently familiar with the phenomena of disease to recognise a symptom when it is described, and to appreciate the necessity for the increasing care, the cleanliness, and the promptitude of action which the author recommends. They cannot but feel their interest in their work deepened by the comprehension they will gain from Dr. Bell's "Notes" of the causes which give rise to the symptoms they have to deal with, and it cannot be doubted that a more thorough and intelligent knowledge of the causes of disease and the possible results to be expected, as well as the actual ones seen, would make the nurse a more efficient co-worker with the doctor than she can be while she only knows the symptoms she sees without understanding the sources from which they arise, and the manner in which they may develope and affect the patient.

* "A Practical Treatise on the Cure of Pulmonary Consumption, with Medicinal, Dietetic, and Hygienic Remedies." By James Weaver, M.D., L.R.C.P., etc. (London: J. and A. Churchill.)

† "Notes on Surgery for Nurses." By Joseph Bell, F.R.C.S. Edin., Consulting Surgeon to the Royal Infirmary, Edinburgh. Edinburgh: Oliver and Boyd. London: Simpkin, Marshall, and Co.

### Vaccination Vindicated.*

It might seem unnecessary at this time of day to put vaccination upon its defence; the services it has conferred on mankind are sufficiently obvious. Students of history know how often and with what virulence epidemics of small-pox once swept over the country, invading impartially the palace and the hovel, and taking from each a terrible toll of deaths. The much-lauded beauties of former days, whose portraits hardly justify the records of their loveliness, were simply those who had escaped the marks of small-pox—whose faces were not "full of great O's," as Shakespeare puts it. It was a rare privilege in those days to be free from the records of the disease, almost as rare as it is now to meet anyone who bears them. These changes cannot be doubted; the only question is whether the decrease in small-pox mortality and the milder character which the disease now bears are due to successful vaccination or to improved sanitation. The contest between vaccinators and anti-vaccinators has raged long, and seems likely to lose nothing for a long time of its force and acrimony. Increased knowledge of the laws of health, greater regard for cleanliness in the person and surroundings, severer restrictions regarding the isolation of patients suffering from infectious disease—all these things tend to lessen the death-rate not only from small-pox, but from all other diseases; and it is one of the favourite arguments of the anti-vaccinators that small-pox has not diminished more than other ailments, and that, indeed its decrease is small compared with that of fevers in general. Dr. McVail, who is a strong and conscientious advocate of vaccination, shows that if small-pox were to be entirely obliterated it could not in the process show so great a decrease as fevers have done; and also that while small-pox mortality has diminished during this century, the deaths from measles and whooping cough have absolutely increased in number. This is a battle of statistics, and a keenly contested one. It would seem to the uninitiated that figures cannot be manipulated, that 2 + 2 will equal 4 to the end of time, and to a certain extent this is the case; but proportions are things very difficult to calculate, and taking a longer or shorter period to judge from may make a serious difference in the averages deduced. It is on this division of the periods in which vaccination, optional and compulsory, has been practised that the two parties in the question differ, while the difficulty of deciding whether vaccination and revaccination has been successful in particular cases, gives a large body of variable evidence which each may claim in turn. But Dr. M'Vail's work shows a thoroughness displayed by no other writer, not even Dr. Alfred Russell Wallace, whose monograph on "Small-pox Statistics and Vaccination" he is most anxious to dispute. His statistics are gathered from the chief countries and cities of Europe, and are vouched for by the best authorities. His tables and diagrams are thoroughly clear and honest, and he makes no attempt to use for his own interest the occasional inaccuracies which will be found in all reports. His arguments are never unfounded, and though he would not claim to be other than a partisan of vaccination, he admits truth on the other side, even when it goes against him. Certainly he can afford to do so. Basing his statements solely on facts which can be verified, he brings forward an amount of evidence in favour of vaccination as a preventive, and still more as a mitigating agent in small-pox, which must make a very forcible impression on all whose eyes are not determinately blinded by prejudice.

The strongest of workers is he who saves for the sake of his children. Marry; the expenses of housekeeping are not to be compared to its gains. All privations become pleasures from the moment that one stints oneself for those one loves.—*Edmond About.*

*Vaccination Vindicated: Being an Answer to the Leading Anti-Vaccinators. By John C. McVail, M.D., D.P.H. Camb., Physician to the Kilmarnock Infirmary; Medical Officer of Health, Kilmarnock; President of the Sanitary Association of Scotland, &c. Cassell and Company (Limited): London and New York.

